# DEVELOPMENT AND CONTENT OF THE STUDY ABROAD COURSE

**DOI:** 10.1093/geroni/igae098.2032

**Published:** 2024-12-31

**Authors:** Elizabeth Fauth

**Affiliations:** Utah State University, Logan, Utah, United States

## Abstract

Pedagogy of faculty-led short study abroad programs posits that these programs offer unique opportunities to build cross-cultural adaptability and global citizenship, expand disciplinary and interdisciplinary content knowledge beyond the traditional classroom, foster experiential learning opportunities, and facilitate personal and professional identity. This presentation on the Aging in a Welfare State covers the how course design, content selection, site visits, assignment, and excursions map onto these objectives. Optimizing three credits (which is ideal for most students’ programs) necessitates reading and assignments before, during and after “in-country” portion of the course. In-country content is delivered by US faculty and Swedish faculty, who purposefully are chosen (or select lecture content) based on their own discipline and expertise. With lecture content in nursing, social work, developmental psychology, gerontology, and more, students from diverse majors often connect closely to at least some of the content within their discipline, but expand learning into new topics. The addition of content on family and child policies is attractive to students outside of gerontology programs. Days are organized around themes, with three lectures in the morning broken up by the Swedish coffee tradition of FIka, a lunch break, and site visits to hospitals, adult day programs, child care programs, senior centers, assisted living facilities, and rehabilitation programs. Site visits offer applied learning experiences, where students can interact with professionals and clients/residents/users. Excursions are used to expose students to Swedish culture, and the historical perspectives that inform their social care policies. Assignments bridge academic application and personal/professional self-reflection.

